# ASSESSING NONSAMPLING BIAS ERROR AMONG SEXUAL MINORITY OLDER ADULTS IN THE CENSUS' HOUSEHOLD PULSE SURVEY

**DOI:** 10.1093/geroni/igac059.462

**Published:** 2022-12-20

**Authors:** Kasim Ortiz

**Affiliations:** University of New Mexico, Albuquerque, New Mexico, United States

## Abstract

Previous research shows data quality and nonsampling bias error reduce accuracy of COVID-19 vaccination coverage in the Census' Household Pulse Survey. However, it is unclear how these issues relate to older sexual minority populations. Moreover, previous research has shown that sexual minority older adults survey nonparticipation rates are similar to their heterosexual older adult peers. Yet, it is less clear whether racial/ethnic variability in nonparticipation rates and patterns of missingness among sexual minority older adults exist. The current investigation utilizes repeated pooled data from the Census' Household Pulse Survey (N=329,078) to fill these gaps. Findings reveal nonsampling bias error contributing to inflated racial/ethnic inequities in vaccination coverage among older sexual minorities, with distinct racial/ethnic variability in missingness patterns among sexual minority older adults. The paper concludes with recommendations for improving survey-based research and details suggestions to improve recruitment strategies that minimize nonsampling bias.

